# N-Doped porous carbons obtained from chitosan and spent coffee as electrocatalysts with tuneable oxygen reduction reaction selectivity for H_2_O_2_ generation†

**DOI:** 10.1039/d3ra02587j

**Published:** 2023-07-27

**Authors:** Alexandra S. M. Wittmar, Thaarmikaa Vigneswaran, Nikola Ranković, Ulrich Hagemann, Nils Hartmann, Ricardo Martínez-Hincapié, Viktor Čolić, Mathias Ulbricht

**Affiliations:** a Lehrstuhl für Technische Chemie II, Universität Duisburg-Essen Universitätsstr. 745141 Essen Germany; b NETZ – NanoEnergieTechnikZentrum, CENIDE – Center for Nanointegration Duisburg-Essen Carl-Benz-Str. 199 47057 Duisburg Germany alexandra.wittmar@uni-due.de; c Electrochemistry for Energy Conversion, Max-Planck Institute for Chemical Energy Conversion Stiftstr. 34-36 45470 Mülheim an der Ruhr Germany, viktor.colic@cec.mpg.de; d Fakultät für Chemie und Biochemie, Ruhr-Universität Bochum Universitätsstraße 150 44801 Bochum Germany; e Interdisciplinary Center for Analytics on the Nanoscale (ICAN), University of Duisburg-Essen Carl-Benz-Str. 199 47057 Duisburg Germany

## Abstract

Nitrogen-containing porous carbons prepared by the pyrolysis of adequate biopolymer-based precursors have shown potential in several electrochemical energy-related applications. However, it is still of crucial interest to find the optimal precursors and process conditions which would allow the preparation of carbons with adequate porous structure as well as suitable nitrogen content and distribution of functional groups. In the present work we suggested a straightforward approach to prepare N-doped porous carbons by direct pyrolysis under nitrogen of chitosan : coffee blends of different compositions and using KOH for simultaneous surface activation. The synthetized carbon materials were tested for the electrochemical oxygen reduction to hydrogen peroxide (H_2_O_2_). A higher fraction of chitosan in the precursor led to a decrease in meso- and nano-porosity of the formed porous carbons, while their activity towards H_2_O_2_ generation increased. The nitrogen species derived from chitosan seem to play a very important role. Out of the synthesized catalysts the one with the largest content of pyridinic nitrogen sites exhibited the highest faradaic efficiency. The faradaic efficiencies and current densities of the synthesized materials were comparable with the ones of other commercially available carbons obtained from less renewable precursors.

## Abstract 2

Nitrogen-doped porous carbons prepared by the pyrolysis of coffee and chitosan have shown potential for electrochemical applications like H_2_O_2_ selective generation.

## Graphical abstract

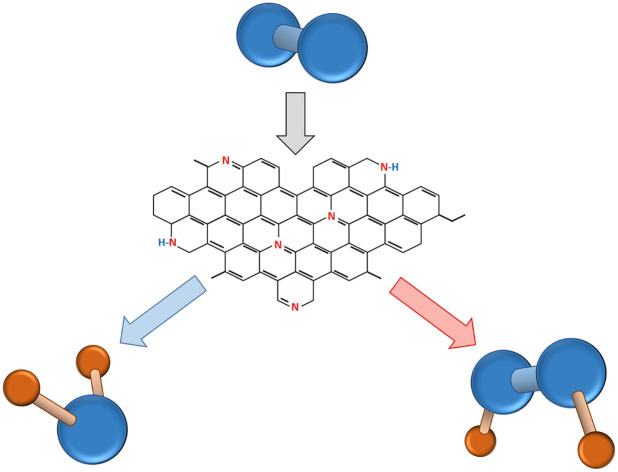

## Introduction

Hydrogen peroxide (H_2_O_2_) is a valuable chemical^1–3^ with a wide variety of applications in industry, which include: pulp and textile bleaching,^4^ wastewater treatment,^5^ biomass upgrading *via* oxidation,^6^ semiconductor cleaning, detergent production, and others.^7^ Additionally, it is used in households around the world as a cheap yet powerful disinfectant, important for combating global pandemics.^8^ H_2_O_2_ has a high oxidation potential in a wide pH range,^9^ making it a suitable oxidant in a wide range of conditions.^4,10^ Consequently, the global production of H_2_O_2_ is steadily rising and has reached 5.5 million tons/year as of 2015,^11^ with a trend of further growth. Currently, H_2_O_2_ is produced on an industrial scale *via* the anthraquinone-mediated reduction of oxygen (O_2_) known as the autooxidation or AO-process for short.^12^ The main drawbacks of the AO-process are the excessive use of organic solvents, its complexity, and organic contamination of H_2_O_2_.^11^ Most use cases of H_2_O_2_, require aqueous solutions of up to 9% by weight.^7^ Alternative routes for H_2_O_2_ have been proposed, such as direct synthesis of H_2_O_2_ from H_2_ and O_2_, thereby eliminating the separation step in production. However, in practice, the gas mixture (H_2_/O_2_) needs to be diluted, in order to avoid the risk of explosion. Moreover, only pure O_2_ in excess can be used, but not air, hence, increasing costs of production and making upscaling difficult, at the current state of the art.^13^ Alternatively, the electrochemical reduction of O_2_ can generate H_2_O_2_ directly, avoiding the aforementioned issues.^14^ The O_2_ reduction reaction (ORR) can proceed *via* the two-electron pathway and generate H_2_O_2_, or alternatively *via* the four-electron pathway and generate water and electricity.^7,15,16^ The two-electron pathway is interesting as it generates H_2_O_2_ in an aqueous solution directly, eliminating the need for the separation of H_2_O_2_ and the risk of explosion.^4^ Recently, it has been demonstrated that with the use of a solid electrolyte, which requires acidic reaction conditions (*e.g.*, Nafion-H®), a 20% by weight aqueous solution of H_2_O_2_ can be obtained.^17^ Such electrochemical devices offer the possibility of onsite production of H_2_O_2_.^7^

Nevertheless, the cost of electrochemical production of H_2_O_2_ is primarily influenced by the amount of electricity consumed per mol of H_2_O_2_. To make electrochemical production of H_2_O_2_ economical, the amount of electricity consumed per mol of H_2_O_2_ produced needs to be minimized.^7^ Cost reduction and efficient use of electricity is possible with a suitable cathode catalyst that is able to achieve high faradaic efficiencies over an extended period and at high current densities.^18,19^ Over the years, many catalyst materials were tested for the electrochemical H_2_O_2_ production, including: pure metal surfaces,^20^ various alloys,^21^ single atom catalysts^22,23^ and carbon-coated metal nanoparticles.^24^ The main issue with noble metal catalysts is their availability. Many are scarce and at the same time difficult to extract and scale.^25^ Additionally, even minute residues of heavy metals, such as mercury^16^ can be problematic in long-term applications in some fields, such as agriculture.

Organic waste and biomass, as nature-derived materials and being low cost and abundant, are recently and intensively studied for clean energy storage and conversion applications.^26^ Carbon materials can be produced from organic waste products, thereby providing a sustainable source of catalyst material.^27^ As the production of municipal waste increases,^28^ the topic of waste recycling and valorisation is crucial for enabling sustainable development.^29^ For instance, the recycling of waste from corn stover,^30^ oats husks,^31^ tire rubber,^32^ eggshells,^33^ sugarcane processing residue,^34^ bamboo^35^ and coffee waste^36–38^ have been considered as possible pathways to produce various carbon materials. Coffee waste, for example, is characterized by high organic content, in the form of polysaccharides, fatty and amino acids, polyphenols and various minerals.^36^ Therefore, the wide consumption of coffee^39^ coupled with its high organic content make it a suitable candidate precursor for carbon materials with variable properties, such as sustainable catalyst materials. Chitin is a naturally abundant polysaccharide, primarily found in crustaceans, with a similar structure and function to cellulose. Chitosan on the other hand, is a *N*-deacetylated derivative of chitin. Both chitin and chitosan possess a higher nitrogen content than synthetically substituted cellulose.^40^ High nitrogen content of chitosan coupled with its biodegradability, biocompatibility, and non-toxicity^41^ make chitosan an excellent starting material to produce nitrogen-doped (N-doped) carbons, especially since it is abundant and a by-product of the food industry.^42^ Recent work conducted by Khan *et al.*, showed that chitosan, in the presence of a co-dopant, in this case graphitic nanoparticles, can enhance the selectivity of the chitosan derived N-doped carbon material towards the two-electron O_2_ reduction and generate peroxide.^43^ Chitosan-derived N-doped electrocatalysts obtained by direct pyrolysis of chitosan or chitosan/1,10-phenanthroline could successfully electrogenerate H_2_O_2_ in amounts high enough for its direct application in Fenton-based electrochemical water treatment.^44^ When such a catalyst is doped with Fe after the first pyrolysis step, a catalyst with improved Fe–N_*x*_ catalytic sites and high selectivity towards the 4e^−^ ORR can be obtained.^45^

In the present work, we studied the influence of the chitosan : coffee ratio of blends and of the biopolymer : KOH ratio used for activation on the porous structure formation and chemical composition of a series of N-doped porous carbons obtained by pyrolysis. The synthetized carbon materials were tested in electrocatalytic processes, namely, the oxygen reduction to H_2_O_2_. The focus was set on developing a facile procedure, which enables the production of high surface area porous carbons which are able to favour the two-electron reaction pathway of the ORR. The complex correlations between the synthesis conditions, material characteristics and their efficiency to the H_2_O_2_ generation were discussed in depth.

## Experimental section

### Materials

Coffee arabica type was obtained from a local supermarket (Kaffee Gold, Markus Kaffee GmbH & Co. KG). Before further use, normal coffee brewing was simulated by using the coffee powder as follows: the mass of coffee filling 5 coffee spoons was inserted in a filter and 1.4 L boiling water were poured in order to extract the soluble matter. The obtained coffee waste was afterwards dried at 100 °C in a laboratory oven and stored dry.

For the preparation of the porous chitosan and of the chitosan : coffee blend spheres, chitosan with medium molecular weight (100 000–300 000 g mol^−1^) from Acros Organics made from the shell of shrimps and crabs *Pandalus borealis* was used as received.

The ionic liquid (IL) 1-butyl-3-methylimidazolium acetate ([Bmim][OAc]) in BASF quality (≥95%) was also purchased from Sigma-Aldrich and used without further purification. Dimethyl sulfoxide (DMSO; analytical reagent; ≥99.5%) from VWR International was used as co-solvent for the polymer dissolution. The solvent mixture used for the biopolymer solutions preparation consisted in [Bmim][OAc] : DMSO = 1 : 1 (wt : wt) ratio.

Other used chemicals include KOH analytical grade (VWR Chemicals) and HCl 37% Ph. Eur. (Carl Roth).

### Preparation of the porous chitosan and chitosan : coffee composites

A chitosan solution with the concentration of 3 wt% was prepared as follows: chitosan was dispersed in a mortar in the IL : DMSO = 1 : 1 mixture. The formed polymer dispersion was transferred to a closed flask and heated at 70 °C until the complete dissolution of the polymer. During the dissolution process the mixture was stirred several times in order to prevent polymer accumulation at the bottom of the flask. For the samples containing coffee, the desired coffee waste amount was homogenously dispersed in the chitosan solution. From the prepared chitosan solution or from the coffee dispersions in chitosan solution the chitosan and the chitosan : coffee spheres were fabricated by dropping *cum* phase separation technique. Drops of about 3 mm diameter were dispensed using a syringe in a water coagulation bath. The obtained spheres were washed several times with water for the complete removal of the solvent. The ionic liquid and DMSO can afterwards be recovered from the coagulation bath.

### Pyrolysis processes

The compositions and procedures shown in Table 1 have been used for the preparation of the N-doped carbons by pyrolysis from coffee waste and chitosan : coffee composites. Ceramic combustion boats were filled with the biopolymer : KOH mixtures and then they were inserted in the middle of the glass tube of the pyrolysis furnace Heraeus Kelvitron S. To create the nitrogen atmosphere, this tube was then connected to the nitrogen circuit and flushed with nitrogen for about 15 minutes. The samples were pyrolyzed at 800 °C for two hours under continuous nitrogen flow. Afterwards the samples are left to cool for two hours under nitrogen to room temperature, the ceramic boat with the carbonized sample was removed from the furnace. For each type of sample 4 of 5 batches were prepared in order to produce enough material. The pyrolyzed materials were washed first with 0.1 N HCl solution in order to remove the KOH residuals and thereafter with deionized water until pH 6 was achieved, then dried and finally stored in closed snap-cap vials until further use.

**Table:** Composition of the precursors used in the pyrolysis processes

Sample name	Biopolymer composition	Biopolymer : KOH ratio	Procedure
S1	100% coffee	1 : 0.5	Mixing^a^
S2	100% coffee	1 : 0.5	Impregnation^b^
S3	Chitosan : coffee = 4 : 1	1 : 0.5	Impregnation
S4	Chitosan : coffee = 2 : 1	1 : 0.5	Impregnation
S5	100% chitosan	1 : 0.5	Impregnation
S6	Chitosan : coffee = 2 : 1	1 : 1	Impregnation

a Mixing = the coffee waste and KOH were milled together in a mortar.

b Impregnation = the desired KOH quantity was dissolved in a small amount of water and the biopolymer was impregnated with this KOH solution, then it was dried in an oven at 100 °C.

### Material characterization

The porosity of the carbonized samples was characterized by nitrogen adsorption using a Coulter SA 3100 surface analyser. Prior to the measurement the samples were degassed for 60 min at 120 °C and after weighing they were degassed again for 600 min at 50 °C. The analysis of the isotherms was done according to Brunauer, Emmett and Teller (BET) and Barrett, Joyner and Halenda (BJH) methods.

Scanning electron micrographs (SEM) of the N-doped carbon powders were recorded with an Apreo S Lo Vac from Thermo Fisher Scientific instrument. The samples were sputtered with Au/Pd (80/20) at 0.1 mbar and 300 mA until a 2–3 nm layer was obtained. Images with different magnifications were taken.

The relative amounts of C, H, N, O in the pyrolyzed samples were determined using a EURO EA Carbon–Hydrogen–Nitrogen–Oxygen (CHNO) elemental analyser from EURO VECTOR. Each sample was measured two times and the presented result represents the average of the two measurements.

The X-ray photoelectron spectroscopy (XPS) measurements were performed with an ULVAC-Phi 5000 Versaprobe II device with monochromatic Al Kα source under 45° angle with respect to the sample surface. The binding energies were corrected using the C 1s peak at 284.5 eV.

Raman spectroscopy measurements were performed using a Renishaw InViva Raman spectroscope with a 532 nm laser over a spectral range from 98 to 3200 Raman shift/cm^−1^.

Dispersions of the prepared carbon materials in 70% isopropanol–30% water have been obtained after treatment for 30 min in a sonication bath. The agglomerate size in dispersion, was determined by dynamic light scattering (DLS) method using a Particle Metrix Stabilizer heterodyne backscattering equipment.

For ToF-SIMS analysis powder materials had to be properly bonded to the sample holder in order to avoid damage of the analyzer. Here, each powder has been fixed on an aluminum plate using a piece of a double-sided adhesive Kapton tape (Plano GmbH). Powders were spread onto the top side of the adhesive tape using a spatula trying to yield a mostly fixed, preferentially closed and flat layer of the material. Prior to mounting the aluminium plates onto the sample holder of the ToF-SIMS instrument, loose powder was removed *via* turning each plate and carefully tapping it with the edges onto a collection tray. For reference measurements a bare Kapton tape has been mounted.

Depth profiling experiments of the surface-near region have been carried out at two distinct sets of parameters employing a ToF-SIMS 5–100 (IONTOF): (i) for qualitative analysis the following ion sources and parameters have been used: the primary ion gun operated in spectrometry mode with Bi^+^ primary ions at 15 kV was scanned in random mode at a field size of 100 × 100 μm^2^ and a digital raster of 128 × 128 pixels. Charge compensation was ensured employing a low energy electron flood gun and O_2_ gas flooding at an oxygen pressure of 2 × 10^−6^ mbar. The analyzer was operated in positive polarity and corrected in order to compensate for surface potential shifts. Depth profiling of the near-surface region *via* non-interlaced sputtering was carried out at an analysis-to-sputtering frame ratio of 1 : 1 using an O_2_^+^ ion source operated at a sputtering voltage of 500 V and a field size of 400 × 400 μm^2^. (ii) For semi-quantitative analysis the following ion sources and parameters have been employed: For analysis Bi_3_^+^ primary ions and O_2_ gas flooding at an oxygen pressure of 4 × 10^−6^ mbar were used. Otherwise the same setting and parameters for the primary ion gun, the analyzer and charge compensation as outlined above were chosen, *cf.* (i) non-interlaced co-sputtering was carried out at an analysis-to-sputtering frame ratio of 1 : 1 using a Cs^+^ and Xe^+^ ion source operated at a Cs^+^ duty cycle of 75%, a sputtering voltage of 500 V and a field size of 400 × 400 μm^2^.

Time-of-flight software corrections have been employed in data analysis. Only those regions with high total secondary ion intensity are selected for analysis, as this results in a significantly improved mass resolution. Also, for analysis of the near-surface region sputter time intervals of (i) 100–600 s and (ii) 100–500 s are chosen in order to account for transient effects in the initial sputtering time window.

### Electrochemical measurements

Unless stated otherwise, all electrochemical measurements were done in a three-electrode electrochemical glass cell with a VSP-3e bipotentiostat (BioLogic, France) and a rotating ring-disk electrode (RRDE) rotator (PINE research model: AFMSRCE, USA). The counter electrode used was a glassy carbon rod (HTW Hochtemperatur-Werkstoffe GmbH), the reference electrode was a home-made reversible hydrogen electrode (RHE), and the working electrode was a RRDE (E6R1; Pine research, USA) consisting of a glassy carbon disk with an area of 0.196 cm^2^ and Pt ring with an area of 0.11 cm^2^. The RRDE had a collection efficiency *N* = 0.25. The electrolyte used was aqueous 0.1 M HClO_4_ prepared from 70% HClO_4_ (ROTIPURAN®Supra) by dilution with Milli-Q water (18.2 MΩ). The catalyst ink was prepared by suspending the catalyst powder in a solution containing 70% isopropanol (99.5%, Roth®), 29.6% Milli-Q water and 0.4% Nafion® (5 wt% Sigma-Aldrich®). The concentration was 2 mg mL^−1^; 10 μL of the dispersion was dropped onto a freshly polished disk and dried under inert atmosphere for 5 min. The catalyst loading in all experiments was 0.1 mg cm^−2^. Since the loading of the carbon catalysts can have a significant effect on their faradaic efficiencies,^46,47^ we have kept the loading constant throughout this manuscript. The solution resistance (*R*_s_) was determined using electrochemical impedance spectroscopy (EIS) from 20 kHz to 1 Hz at the open circuit voltage with a sinus amplitude of 5 mV. The Nyquist plots and equivalent circuit used to analyse the pot are given in the ESI (Fig. SI4†).

Before ORR activity was investigated, the Pt ring of the RRDE was electropolished with cyclic voltammetry (CV) by sweeping the potential between 0.059 and 1.5 V at a scan rate of 200 mV s^−1^ under argon saturation (Ar 5.0, Air Liquide) for 50 cycles. The catalyst surface deposited on the working electrode was pre-treated in an argon saturated solution with 5 cycles of CVs from 0 to 0.9 V. Thereafter, the electrolyte was purged with oxygen (O_2_, 4.0, Air Liquide) for 15 min before linear sweep voltammograms (LSVs) were recorded at a scan rate of 10 mV s^−1^, and a rotation speed of 1600 rpm from open circuit voltage to −0.2 V. To detect the H_2_O_2_ produced during ORR the Pt ring potential was fixed at 1.1 V. The faradaic efficiency (FE [%]) was calculated according to relation (eqn (1)):1
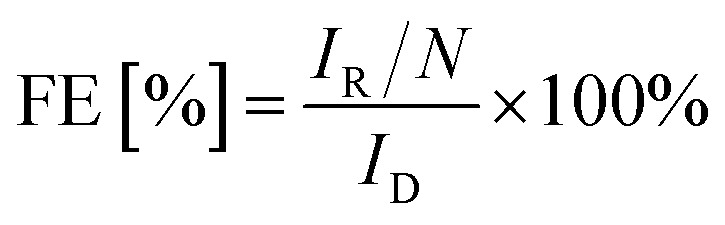
*I*_R_ denotes the ring current, *I*_D_ the disk current and *N* the collection efficiency (0.25). The partial current density (*j*_H_2_O_2__) was calculated as follows (eqn (2)):2
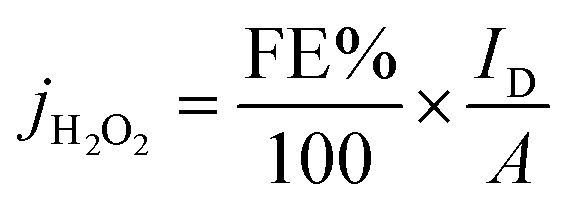
*A* denotes the geometric surface area of the disk (0.196 cm^2^), all other terms have their usual meaning.

## Results and discussion

**Fig. 1:**
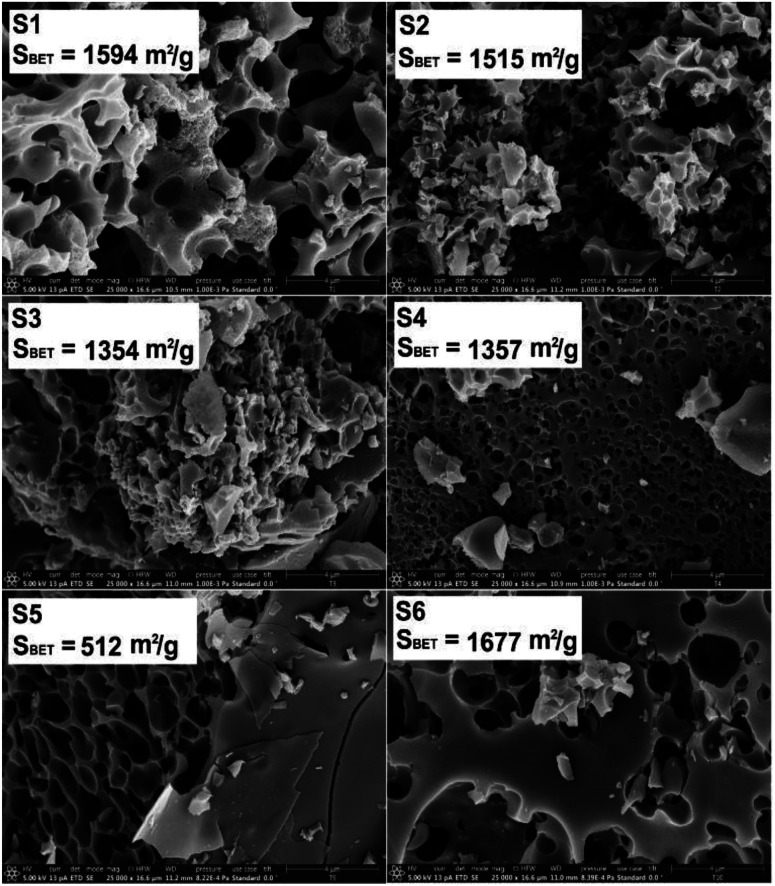
SEM micrographs and the specific surface area (from BET analysis) for the porous carbon-based samples. Note, the scale bars at the bottom right of the SE micrographs are 4 μm.

**Fig. 2:**
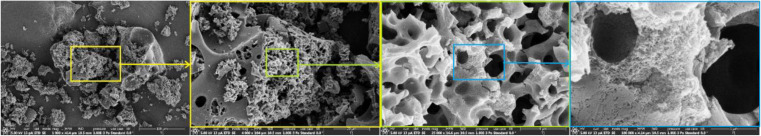
SEM micrographs at different magnifications for the sample S1. Note, the scale bars at the bottom right of the SE micrographs from left to right are 300, 20, 4 and 1 μm.

The SEM micrographs of the carbon-based materials (Fig. 1) show that the biochars are constituted from larger chunks which exhibit an extensive network of interconnected macropores and some smaller particles. By increasing the magnification, as exemplified for the sample S1 (Fig. 2), it was observed that in addition to the macropores the bulk of the materials also possesses a network of meso- and nanopores which are responsible for the very high specific surface areas. With the increase of the chitosan ratio in the used precursor mixture, the proportion of the meso- and nanopores present in the carbonized materials decreases, a fact which agrees with the values of the measured specific surface areas (*S*_BET_ ≈ 1500 m^2^ g^−1^ for pyrolyzed coffee, *S*_BET_ ≈ 1350 m^2^ g^−1^ for pyrolyzed chitosan/coffee mixtures and *S*_BET_ ≈ 500 m^2^ g^−1^ for pyrolyzed chitosan). These differences in the specific surface areas of the carbonized materials may also be a consequence of the differences in the porous structures of the biopolymeric precursors used: while the porous chitosan obtained by freeze-drying from hydrogels has generally a microporous cellular structure,^48^ the coffee waste, though more porous, exhibits a poorly developed mesoporosity.^49^ A higher KOH ratio in the precursor mixture seems to favour the formation of even higher specific surface areas. This agrees with the findings of Tseng *et al.*,^50^ who were able to demonstrate that by increasing the KOH/char ratio when a chemical activation is performed the micropore development will be favoured, leading to the formation of carbons with higher specific surface areas.

The agglomerate size in 70% isopropanol–30% water, relevant for the electrode preparation, was estimated to be between *ca.* 300 and 600 nm (see ESI Fig. SI5†).

**Fig. 3:**
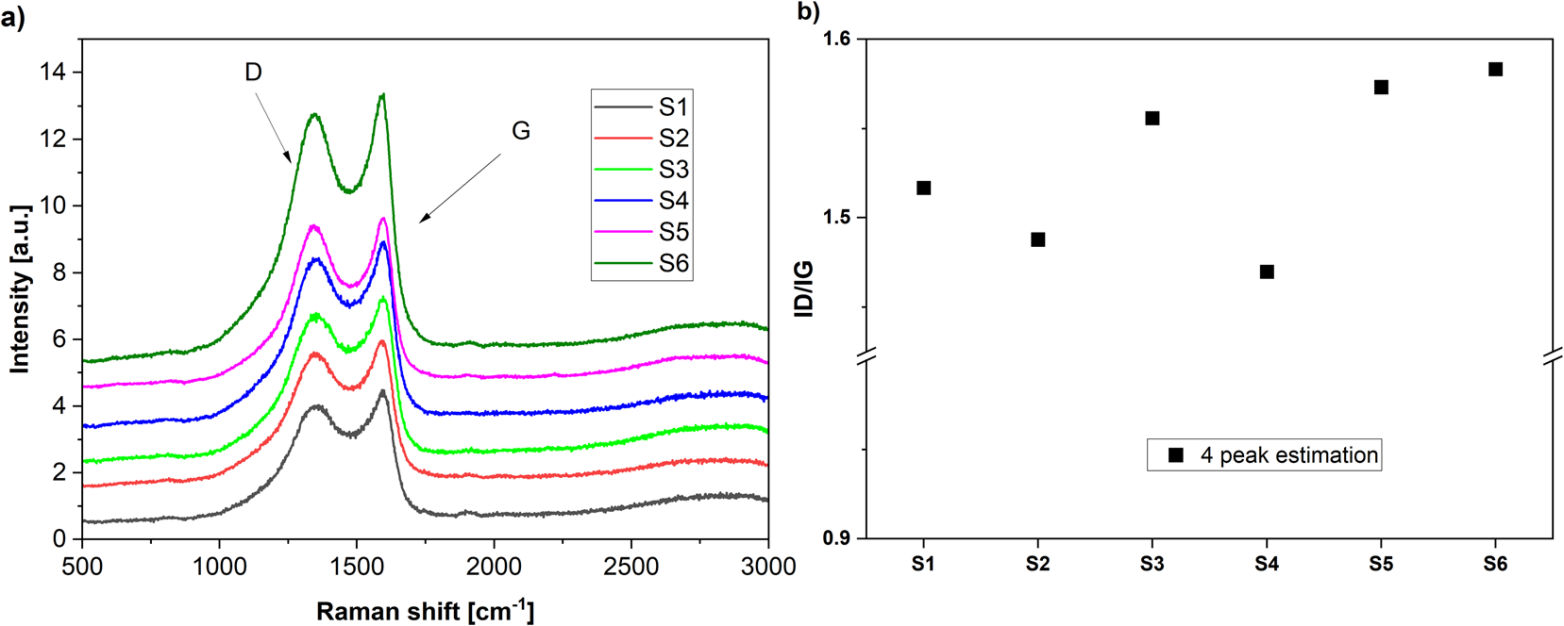
(a) Raman spectra of the various carbons obtained in the present work. (b) The calculated *I*_D_/*I*_G_ ratios by 4 peak fitting.

The Raman spectra of the N-doped carbons are presented in Fig. 3. All spectra contain the D and G peaks which are specific to the sp^2^ carbons.^51^

The D band is assigned to the disordered type carbon and the G band corresponds to the sp^2^ hybridized carbon in the graphite lattice, thus, the intensity ratio *I*_D_/*I*_G_ gives a measure to the disorder degree present in the carbon lattice.^52^

The disorder degree in the carbon lattice seems to be related to the nitrogen content in the carbon-based materials (Table 2). The samples with the highest nitrogen content (S5) and (S3) also exhibit the highest disorder degree. Exception seems to be the sample (S6) which exhibits high disorder but the nitrogen content is much lower. However, deconvolution of the broad signal peaks into multiple strongly overlapping bands can be done only with considerable statistical uncertainty, so these values need to be taken tentatively.^53^

**Table:** Elemental composition of the samples pyrolyzed 2 h at 800 °C, determined by CHNO and XPS analysis

Sample	C [%]		N [%]		O [%]	
	CHNO	XPS	CHNO	XPS	CHNO	XPS
S1	74.9	70.7	0.7	1.7	—	22.5
S2	77.5	84.7	0.2	1.3	—	13.0
S3	74.9	84.1	3	3.5	—	10.0
S4	67.7	65.6	0.7	1.6	—	24.9
S5	67	86.8	3.8	3.5	19.2	9.0
S6	57.9	68.2	1.7	1.9	20.1	23.2

Clearly, the nitrogen amount in the pyrolyzed materials is strongly dependent on the used precursors: the pyrolyzed chitosan contains the highest amount of nitrogen (Table 2). For some of the samples the oxygen could not be detected by CHNO method but was observed by XPS suggesting that there may be oxygen in the sample bound in complexes, which may be present in the samples as impurities. Note, the ratio of chitosan : coffee of the biopolymer used for the preparation of sample S3 is 4 : 1, *i.e.*, higher than those used for sample S4, that is, 2 : 1, *cf.*Table 1.

**Fig. 4:**
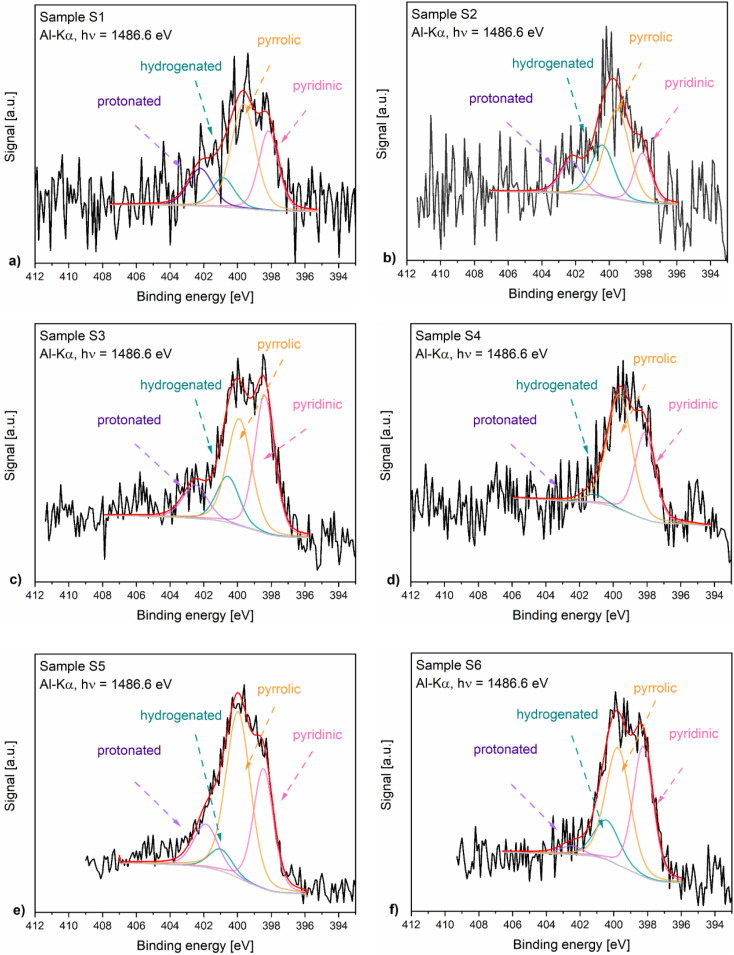
N 1s XPS spectra of samples S1 (a) to S6 (f).

**Fig. 5:**
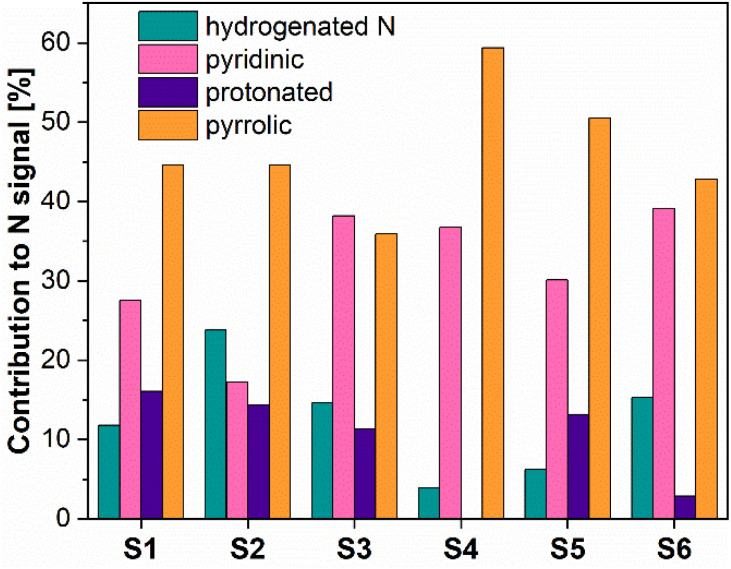
Nitrogen species distribution for the N-doped porous samples, obtained from XPS.

Fig. 4 presents the N 1s XPS spectra of the nitrogen-doped carbons obtained in the frame of this work. As the position and the intensity of the N 1s peaks of the nitrogen-doped carbons prepared by thermal decomposition of biopolymer-based precursors may be affected by different configuration effects like hydrogenation, oxidation or protonation, the deconvolution of the spectra and the assignment of the peaks is difficult. The recorded spectra exhibited peaks around 398.5 eV, 400.2 eV, 400.6 eV and 402 eV which based on specialized literature assignments^54,55^ and according to our previous results^56^ were ascribed to the pyridinic nitrogen, pyrrolic nitrogen, hydrogenated nitrogen and protonated nitrogen, respectively. Additionally, the distribution of the nitrogen species within the analysed N-doped carbons is shown in Fig. 5.

The XPS results confirmed that the materials prepared from precursors with a higher chitosan content contained higher amounts of total nitrogen trapped inside the carbon lattice. The relationship between certain nitrogen species and catalyst performance is discussed later in this work.

Since it is well-known that the presence of trace amounts of metals can influence the selectivity of carbon-based catalysts for the ORR,^57–59^ time-of-flight secondary ion mass spectrometry (ToF-SIMS) has been employed in order to analyze traces of transition metals within the catalytic powders, which, *e.g.*, could stem from the natural precursor materials coffee and chitosan or from the preparation procedure in general,^60,61^ ToF-SIMS in this respect offers a high surface and detection sensitivity, which makes it well suited for ppm level metal analysis of surfaces and near-surface layers.

At first, near-surface analysis of samples S1 to S6 employing an O_2_^+^ ion source for sputtering has been performed. This ensures a particularly high sensitivity for the detection of electropositive elements, such as transition metals.^62^ As the matrix elements of the samples result in a rich and complex mass spectrum, the analysis here focuses on the lower mass range, *i.e.*, on metals from the first transition series, in order to ensure proper peak identification. Note, this includes common trace elements in coffee and chitosan as reported in the literature.^60,61^Table 3 lists respective secondary ion intensities normalized by the intensity of the C^+^ mass peak. Virtually no mass peaks of metals from the first transition series were detected on the Kapton tape reference sample.

Normalized secondary ion intensities of N^+^, Si^+^, M^+^ mass peaks

**Table:** 

Sample	*I*_N^+^_/*I*_C^+^_	*I*_Si^+^_/*I*_C^+^_	*I*_Ti^+^_/*I*_C^+^_	*I*_V^+^_/*I*_C^+^_	*I*_Cr^+^_/*I*_C^+^_
S1	0.0092	40	0.25	0.0054	0.011
S2	0.0066	45	0.05	0.0031	0.003
S3	0.0217	37	0.27	0.0051	0.039
S4	0.0084	105	0.50	0.0028	0.037
S5	0.0187	31	0.22	0.0033	0.061
S6	0.0115	102	1.17	0.0094	0.075

**Table:** 

Sample	*I*_Mn^+^_/*I*_C^+^_	*I*_Fe^+^_/*I*_C^+^_	*I*_Ni^+^_/*I*_C^+^_	*I*_Co^+^_/*I*_C^+^_	*I*_Cu^+^_/*I*_C^+^_
S1	0.074	1.45	0.034	0.0366	0.83
S2	0.033	0.82	0.011	0.0022	0.64
S3	0.071	1.07	0.021	0.0073	0.07
S4	0.107	0.76	0.010	0.0038	0.14
S5	0.015	1.20	0.017	0.0042	0.05
S6	0.114	1.47	0.017	0.0028	0.18

For further reference also normalized intensities of the N^+^ and Si^+^ mass peaks are shown in Table 3. These ion intensities show a reasonable linear relationship to the corresponding atomic ratios obtained from the XPS analysis (Table SI2†), suggesting that matrix effects, which in many cases complicate the ToF-SIMS analysis, are weak.^62,63^ For this reason, the normalized secondary ion intensities here, in first approximation, are considered to represent a measure of the relative concentration ratios. In good agreement with the XPS data, the normalized intensity of the N^+^ mass peak shows an increasing trend with increasing chitosan content of the precursor material. As evident from the data, however, the normalized intensity of the mass peaks of most transition metals shows no clear correlation with the coffee to chitosan mixture ratio. One notable exception being the normalized intensity of the Cr^+^ mass peak, which increases with increasing chitosan content of the precursor material in S2–S5. Note, samples S2–S5 are prepared following the same overall procedure, *cf.*Table 1, hence no matrix effects as a result from varying preparation conditions are expected. Also, the samples prepared from 100% coffee, S1 and S2, yield a higher normalized intensity the Cu^+^ mass peak, when compared with S5, prepared from 100% chitosan, and S3, S4 and S6, prepared from material containing higher amounts of chitosan.

For semiquantitative MCs^+^ analysis complementary measurements employing a Cs^+^/Xe^+^ ion source has been carried out.^64^ This yields MCs^+^ secondary ions of the transition metals, M. Also Si in this analysis yields SiCs^+^ secondary ions. Respective secondary ion intensities of the SiCs^+^ mass peak are used for normalization. Generally, *I*_MCs^+^_/*I*_SiCs^+^_ intensity ratios are less affected by matrix effects and hence scale with corresponding relative concentration ratios M/Si.^64^ Unambiguous peak identification, however, is challenging in the higher mass range. Also mass interferences limit the analysis. The FeCs^+^ mass peak, for example, overlaps with the Si_2_Cs^+^ mass peak. Isotope patterns can help to identify such mass interferences and allow for proper peak identification. The intensities of SiCs^+^, ^29^SiCs^+^, ^30^SiCs^+^ mass peak, for example, are in good agreement with the natural isotope pattern of Si, which clearly allows one to identify the SiCs^+^ mass peak. Mass interferences, however, cannot generally be discerned and ruled out in the high mass range, particularly for monoisotopic elements, *e.g.*, Mn and Co. For these reasons *I*_MCs^+^_/*I*_SiCs^+^_ intensity ratios here are considered as upper bounds. In order to calculate upper bounds of the concentration of the transition metals these intensity ratios are multiplied with the atomic concentration of Si from the corresponding XPS analysis, *i.e.* about 1 at%. Table 4 lists respective upper bounds of the transition metal concentrations for samples S2 and S5, which are prepared from 100% coffee and 100% chitosan, respectively. Most values are in the range 10–1000 ppm range. The upper bounds for Mn are close to 1 at%. No Mn is detected in the XPS analysis suggesting, as discussed above, that the Mn^+^ mass peak indeed significantly interferes with mass peaks of other secondary ion species, *e.g.*, CsKO^+^.

**Table:** Upper bounds of the concentration of transition metals (ppm)

Sample	Ti	V	Cr	Mn	Fe	Ni	Co	Cu
S2	225	94	107	3080	771	525	70	565
S5	87	225	201	7260	680	465	17	750

As expected, ToF-SIMS analysis of the near-surface region of the catalytic materials reveals the presence of some metals of the first transition series, *i.e.*, Ti, Cr, Fe, Ni and Cu are identified *via* their isotope patterns. Normalized intensities of the N^+^ and Si^+^ mass peaks show a reasonable linear relationship to the corresponding atomic ratios obtained from the XPS analysis, suggesting that (i) matrix effects are weak and (ii) normalized secondary ion intensities represent relative measures of the concentration ratios. In good agreement with the XPS data, the normalized intensity of the N^+^ mass peak shows an increasing trend with increasing chitosan content of the precursor material. Also, the normalized intensity of Cr^+^ mass peak correlates with the chitosan amount used in the precursor material and the normalized intensity of the Cu^+^ mass peak in the samples prepared from 100% coffee is significantly higher when compared with samples prepared from 100% chitosan. Otherwise no clear correlation between the normalized intensity of transition metal secondary ion mass peaks is evident from the data. Upper bounds of the concentration of transition metals as obtained from MCs^+^ measurements generally are in the range 10–1000 ppm. For Mn an upper bound ≫ 1.000 ppm is calculated. The Mn concentration, however, is expected to be well below this value, as no Mn is detected in the XPS measurements.

For further evaluation and best illustration, Fig. SI7A–I† show correlation graphs, plots of the measured faradaic efficiency *vs.* the normalized metal secondary ion intensities *I*_M^+^_/*I*_C^+^_. We would like to note, that, the thus obtained data mostly do not seem to show a direct correlation with the faradaic efficiency. One exception being the data for Cr. The samples, however, appear to contain only very low amounts of Cr, *cf.*Table 4, *i.e.*, when compared with the concentration of nitrogen groups, such as pyridinic nitrogen, *cf.*Fig. 5.

**Fig. 6:**
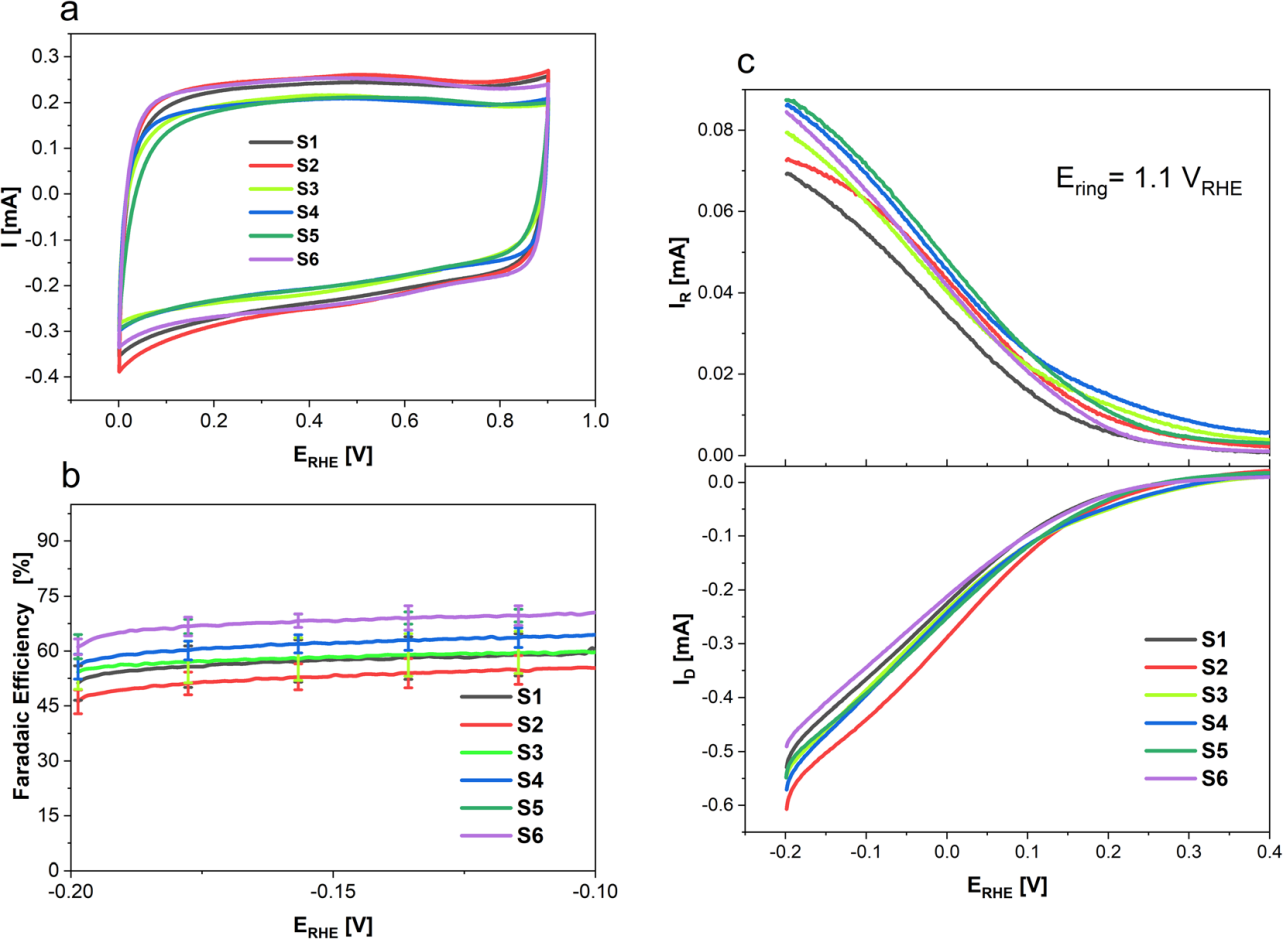
Linear sweep voltammograms in 0.1 M HClO_4_ at 10 mV s^−1^ and 1600 rpm where (a) shows cyclic voltammograms in Ar-saturated 0.1 M HClO_4_ from 0 to 0.9 V before ORR, (b) shows the FE as a function of electrode potential in O_2_-saturated 0.1 M HClO_4_ and (c) shows the ring and disk currents in O_2_-saturated 0.1 M HClO_4_.

To determine the selectivity of N doped porous carbons towards 2-electron oxygen reduction to H_2_O_2_ rotating ring-disk electrode (RRDE) measurements were conducted and the results are presented in Fig. 6. Where Fig. 6a shows the CVs of each sample in an Ar saturated electrolyte from 0 to 0.9 V recorded at 100 mV s^−1^, done as a preconditioning step. Additionally, the CVs in the non-faradaic region allow the determination of the double layer capacitance (*C*_dl_) of the various samples. While S1, S2 and S6 show higher surface areas (*S*_BET_) than S4, S3 and S5 (*cf.*Fig. 1), the CV response and *C*_dl_ are the same order of magnitude for all materials (Fig. SI1 and SI2†). To determine the selectivity of N-doped porous carbons towards two-electron ORR to H_2_O_2_ RRDE measurements were conducted. Linear sweep voltammetry was used to study H_2_O_2_ formation in a O_2_ saturated electrolyte; the corresponding disk current which arises from the O_2_ reduction is shown in Fig. 6c (bottom), whereas the Pt ring current originates from oxidation of H_2_O_2_ (Fig. 6c, top). The faradaic efficiency of N-doped porous carbons at various potentials is presented in Fig. 6b. Out of the N-doped porous carbons tested, sample S6 showed the highest FE of 69 ± 2% at −0.2 V. This value is nearly 15% higher than that for samples S1 and S2 which were derived only from coffee (*i.e.*, no chitosan used). Such a difference in the FE of the conversion to H_2_O_2_ suggests the importance of chitosan doping in the synthesis of carbon catalysts for H_2_O_2_ generation. Furthermore, the highest ring currents were achieved by S5, S4, S6 and S3, whereas lower ring currents were generated by S1 and S2.

**Fig. 7:**
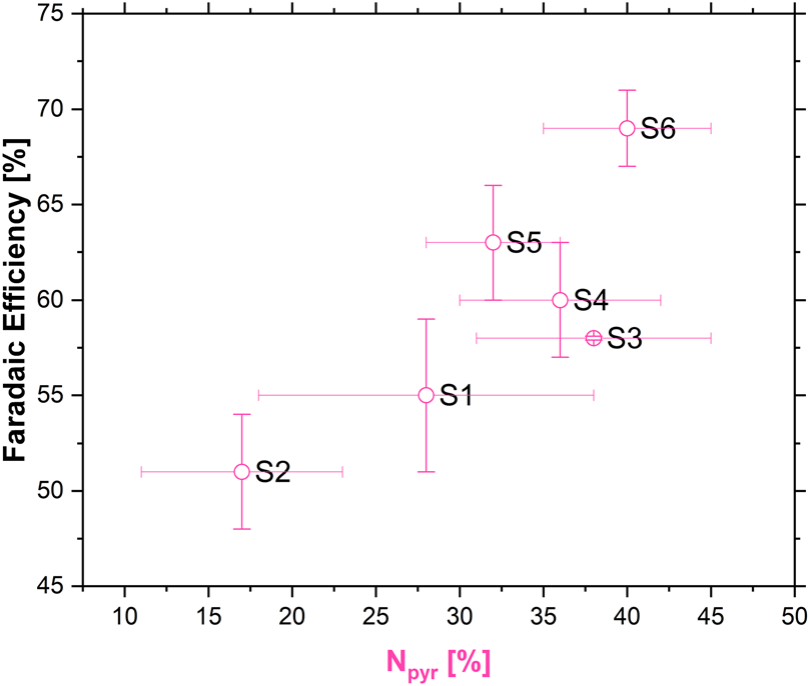
Correlation between pyridinic nitrogen content (from XPS) and FE [%].

The existence of multiple nitrogen functional groups in the N-doped carbon material makes it difficult to decouple which catalytic sites are active during catalysis, and moreover under which conditions.^65,66^ Nonetheless, a quasi linear relationship between the amount of pyridinic nitrogen in our carbons and FE [%] was obtained as shown in Fig. 7.

Effects of different nitrogen groups on the ORR have been studied with computational methods over the years, in an attempt to decouple the various effects different nitrogen groups have on the ORR. However, the focus of these studies was usually the four-electron pathway, and only recently the two-electron pathway has attracted more interest by researchers.^67,68^

Recent experimental work done by the groups of Strasser^69,70^ and Fornasiero^71^ has tried to shed more light on the complicated interface during ORR. By measuring the nitrogen content of the carbons before and after the ORR at different pH, it was observed that the amount of pyridinic nitrogen diminishes after the ORR in low pH. This observation was explained by a protonation effect that occurs on the pyridinic nitrogen atom which influences the ORR pathway, thus making pyridinic nitrogen active for the ORR to H_2_O_2_ in acidic pH, but not in neutral or basic pH, where other nitrogen functional groups have a more pronounced effect on the ORR pathway.^69–71^

Depending on the effective catalytic activity of pyridinic nitrogen on one hand and metal-containing structural entities on the other hand transition metals might well contribute to the overall catalytic performance of the samples.^57,72^ In view of the higher concentration of pyridinic nitrogen these species could dominate the catalytic performance of the samples prepared with the addition of chitosan. In addition, transition metals might explain the catalytic performance of those samples prepared from 100% coffee.

Table 5 summarizes the faradaic efficiencies and partial current densities of all tested samples at −0.2 V. Again, it is shown that the carbon samples that contained chitosan as a nitrogen source show higher faradaic efficiency. The faradaic efficiency might also correlate with the degree of disorder (*cf.*Fig. 3); a higher degree of disorder of the carbon lattice could lead a higher faradaic efficiency. Additionally, higher specific surface area suggests an improved accessibility of the active centres, which may also lead to improved electrocatalytic activity. On the other hand, the partial current densities per geometric surface area, *j*_H_2_O_2_,geom_ which were calculated from eqn (2) do not show significant differences. When normalized per electrochemical surface area (ECSA) gained form the double layer charging experiments (Table S1†), *j*_H_2_O_2_,DL_, we observe that S5 has a markedly higher partial current density, notably this carbon also has a low BET surface area.

**Table:** Faradaic efficiency and the partial current densities for H_2_O_2_ generation at −0.2 V of porous carbon-based samples

Sample	FE @ −0.2 V [%]	*j*_H_2_O_2_,geom_ @ −0.2 V [mA cm^−2^]	*j*_H_2_O_2_,DL_ @ −0.2 V [μA cm^−2^]	*S*_BET_ [m^2^ g^−1^]
S1	55 ± 4	1.48	2.92	1594
S2	51 ± 3	1.70	2.74	1515
S3	58 ± 0.1	1.68	3.74	1354
S4	60 ± 3	1.84	3.00	1357
S5	63 ± 3	1.85	5.46	512
S6	69 ± 2	1.70	3.87	1677

While most applications of hydrogen peroxide require diluted solutions,^73,74^ it is still desirable to be able to achieve higher concentrations in real-world devices. However, this is highly dependent on the design of the cell.^75^ To gain insight into the long-term performance of our porous carbon materials we conducted bulk electrolysis experiments in a home-made H-cell on a gas diffusion layer as the substrate electrode (see ESI; Fig. SI6†). Similarly, to our results in the three-electrode set up, samples S6 and S5 showed the best performance, with 1662.5 μmol mg_cat_^−1^ cm^2^ h^−1^ and 1757.5 μmol mg_cat_^−1^ cm^2^ h^−1^ of H_2_O_2_ produced, respectfully (Fig. SI3†). The slight difference in total amount of H_2_O_2_ produced per catalyst loading could be explained by a difference in the electrochemical surface area of the samples.

The inversely proportional dependence of the selectivity for the two-electron pathway of *S*_BET_ is at first glance in contrast to some previously observed effects for commercial carbon black materials, where micropore volume promoted the reaction^18^ (although it is well-known that mesopores also display a promoting effect^76^). However, the carbons investigated in that work did not contain a substantial amount of nitrogen groups, that is *ca.* 50–2000 ppm only, while the best performing carbons in this work contain several at% N (*cf.*Table 2), *i.e.*, several orders of magnitude more. In this regard special attention needs to be paid to the nitrogen groups introduced by the addition of chitosan. The exact role of different nitrogen functionalities in carbon are still somewhat under debate.^19^ This is due to several factors, such as: the influence of even minor metal contamination,^77,78^ effects of loading,^79^ hydrophobicity,^80^ difficulty synthesizing carbons with a single site type, differences between as-synthesized materials and materials in operando, *etc.* Zhao *et al.*^81^ showed that the overall amount of N does not in itself determine ORR activity, but that pyridinic and quaternary N lead to a lower overpotential for ORR, although in their work they used the RDE and only observed the overall ORR. Sharifi *et al.*^82^ found that the four-electron process proceeded mostly on quaternary N, while pyridinic sites favoured the two-electron pathway on heat-treated N-doped multiwalled carbon nanotubes. Several other researchers also noted the positive effect of pyridinic nitrogen to improve the selectivity for the 2-electron process.^10,71^ DFT calculations by Chen *et al.*^83^ identify several types of graphitic-N sites as active for the four-electron process. Nonetheless, *e.g.*, Artyushkova *et al.*^54^ have considered that pyrrolic N serves as an active site for the first step of the oxygen reduction reaction, while it was assumed that pyridinic N reduces H_2_O_2_ further to water but in a lower amount.

## Conclusions

We observed that chitosan leads to a decrease in meso- and nano-porosity in pyrolyzed carbon materials obtained from composites with coffee waste. RRDE measurements, in turn, showed that the samples prepared with chitosan showed higher activity towards H_2_O_2_ generation. The best faradaic efficiency of the sample with the highest pyridinic nitrogen content seems to be the result of several favourable circumstances, such as nitrogen presence in the carbon lattice, relatively high content of the pyridinic species and a higher specific surface area. To summarize, we used an abundant, easily accessible and separable waste source to synthesize carbon materials of tuneable properties capable of promoting the two-electron ORR. The faradaic efficiencies and current densities are comparable to many commercial carbon materials,^56^ which are often obtained from less “green” sources, *e.g.*, by the partial combustion of aromatic oils, coal char and ethylene from fossil fuel sources.

## Author contributions

Alexandra S. M. Wittmar: supervision, conceptualization, investigation, formal analysis, validation, writing – original draft and introduction. Thaarmikaa Vigneswaran: investigation, formal analysis, validation. Nikola Ranković: investigation, formal analysis, validation, writing – original draft and introduction. Ulrich Hagemann: XPS & Raman spectroscopy: investigation, formal analysis**,** writing – review & editing Nils Hartmann: TOF-SIMS: investigation, formal analysis, writing. Ricardo Martínez-Hincapié: supervision, conceptualization, formal analysis, writing – review & editing. Viktor Čolić: conceptualization, supervision, writing – review & editing. Mathias Ulbricht: supervision, writing – review & editing.

## Conflicts of interest

The authors declare that they have no conflicts of interest.

## Supplementary Material

RA-013-D3RA02587J-s001
