# Supplementary material for: N-Doped porous carbons obtained from chitosan and spent coffee as electrocatalysts with tuneable oxygen reduction reaction selectivity for H2O2 generation
Source: RSC Adv. 2023 Jul 27;13(33):22777–88. doi: 10.1039/d3ra02587j (PMC10372475; doi:10.1039/d3ra02587j)
Supplement: RA-013-D3RA02587J-s001 [file RA-013-D3RA02587J-s001.pdf]

Electronic Supplementary Material (ESI) for RSC Advances.
This journal is © The Royal Society of Chemistry 2023

N-doped porous carbons obtained from chitosan and spent coffee as electrocatalysts with tuneable oxygen reduction reaction selectivity for H_2O_2 generation

Supporting Information

Alexandra S. M. Wittmar[a,b,*], Thaarmikaa Vigneswaran[a], Nikola Ranković[c,d], Ulrich Hagemann[b,e], Nils Hartmann,[b,e] Ricardo Martínez-Hincapié[b,c], Viktor Čolić[b,c,*] and Mathias Ulbricht[a,b]

[a]*Lehrstuhl für Technische Chemie II, Universität Duisburg-Essen, Universitätsstr. 7, 45141 Essen, Germany.*

[b]*NETZ – NanoEnergieTechnikZentrum, CENIDE – Center for Nanointegration Duisburg-Essen, Carl-Benz-Str. 199, 47057 Duisburg, Germany.*

[c]*Electrochemistry for Energy Conversion, Max-Planck Institute for Chemical Energy Conversion, Stiftstr. 34-36, 45470 Mülheim an der Ruhr, Germany*

[d]*Fakultät für Chemie und Biochemie, Ruhr-Universität Bochum, Universitätsstraße 150, 44801 Bochum*

[*]*Corresponding authors*

Determination of the electrochemically active surface area (ECSA): A procedure was followed in accordance with [1]. In short, cyclic voltammetry curves (CV) were recorded in a non-Faradaic potential window in an argon saturated 0.1M $HClO_4$ solution. The CVs were recorded at five different scan rates (Figure SI1). After witch the anodic current at the middle of the potential window was plotted against the scan rate (Figure SI2). A linear fit of these data gives the capacitance of the double layer (C_{dl}). This value was divided by the specific capacitance of carbon basal plane (C_s=20 µF cm^{-2}), a value commonly used in literature to get the ECSA [2].

Gas Diffusion Electrode preparation: A catalyst ink solution (2 mg/mL) of the carbon materials was prepared in the exact same way as described in the methods section of the paper. A Gas Diffusion Layer (GDL) was used as the substrate electrode (Freudenberg H15, Quintech). The GDL was cut into a cycle with a diameter of 22 mm and a geometrical surface area of 3.8 cm^2. The GDL was dried over night at 110 °C, before the catalyst ink was deposited on the surface of the GDL to make the Gas Diffusion Electrode (GDE). 190 µL of the catalyst ink solution (2 mg/mL) was deposited using a micro pipette in five increments of 38 µL to achieve a catalyst loading of 0.1 mg/ cm^2 ±0.02 mg/cm^2. After each increment of catalyst ink was deposited the GDL was dried in the oven at 110 °C for 2 minutes, after the last increment was deposited the now made GDE was kept in the desiccator before use.

H-Cell: Bulk electrolysis experiment were conducted in a home-made H-cell, a picture and a scheme of the setup is shown in Fig. SI6. Nafion 117 membrane (20 mm diameter) was used as the separator. Before use the membrane was activated by first boiling in 5% H_2O_2 for one hour, then boiling in 0.5 M H_2SO_4 for one hour and finally boiling in MQ-water. After activation the membrane was kept in ultrapure water until use and never left to dry out. Both cathodic and anodic compartments were charged with 100 mL of 0.1 M $HClO_4$.

H_2O_2 quantification: The H_2O_2 quantification was done with permanganometry. To prepare and standardize the 0.1 N (0.02 M) solution of $KMnO_4$ the following procedure was used. 3.2-3.25 g of $KMnO_4$ was weighed out and transferred into a beaker, 1 L of ultrapure water was added to dissolve the solid. The solution was then boiled for 30 minutes and left to cool to room temperature thereafter. Afterwards, the solution was filtered through a sintered glass funnel. The filtrate was collected in a clean vessel and stored away from direct sunlight.

The solution was then standardized with sodium oxalate ($Na_2C_2O_4$) as follows. 0.3 g of dried sodium oxalate was weighed into an Erlenmeyer flask, 200 mL of ultrapure water was added along with 50 mL of sulfuric acid and ultrapure water solution (1:3). The flask was heated to boiling on a hot plate, after which the solution was titrated with $KMnO_4$ from a 50-mL Class-A burette (VWR Chemicals) until the appearance of a faint pink colour that persisted for 30 seconds [3]. The concentration was calculated from equation SI1.

$$C(KMnO_4) = \frac{2 \times m\,(Na_2C_2O_4)}{5 \times M\,(Na_2C_2O_4) \times V(KMnO_4)}$$

(SI1)

The solution of $KMnO_4$ used in this work had a normality of 0.095 N (0.019 M). The solution was periodically re-standardized and no deviations in concentration was observed.

To determine the concentration of H_2O_2 an aliquot of 5 mL was pipetted from the cathodic compartment of the H-cell into a glass vial with a Teflon stirring rod and 1 mL of 0.5 N H_2SO_4 was added. The 0.095 N solution of $KMnO_4$ was added with a micro pipette in 10 µL increments with constant stirring until the appearance of a faint pink colour that persisted. The amount of mols of H_2O_2 was calculated using equation SI2.

$$n(H_2O_2) = \frac{5}{2} \times C\left(MnO_4^-\right) \times V\left(MnO_4^-\right)$$

(SI2)

Comment [MOU]: Please check

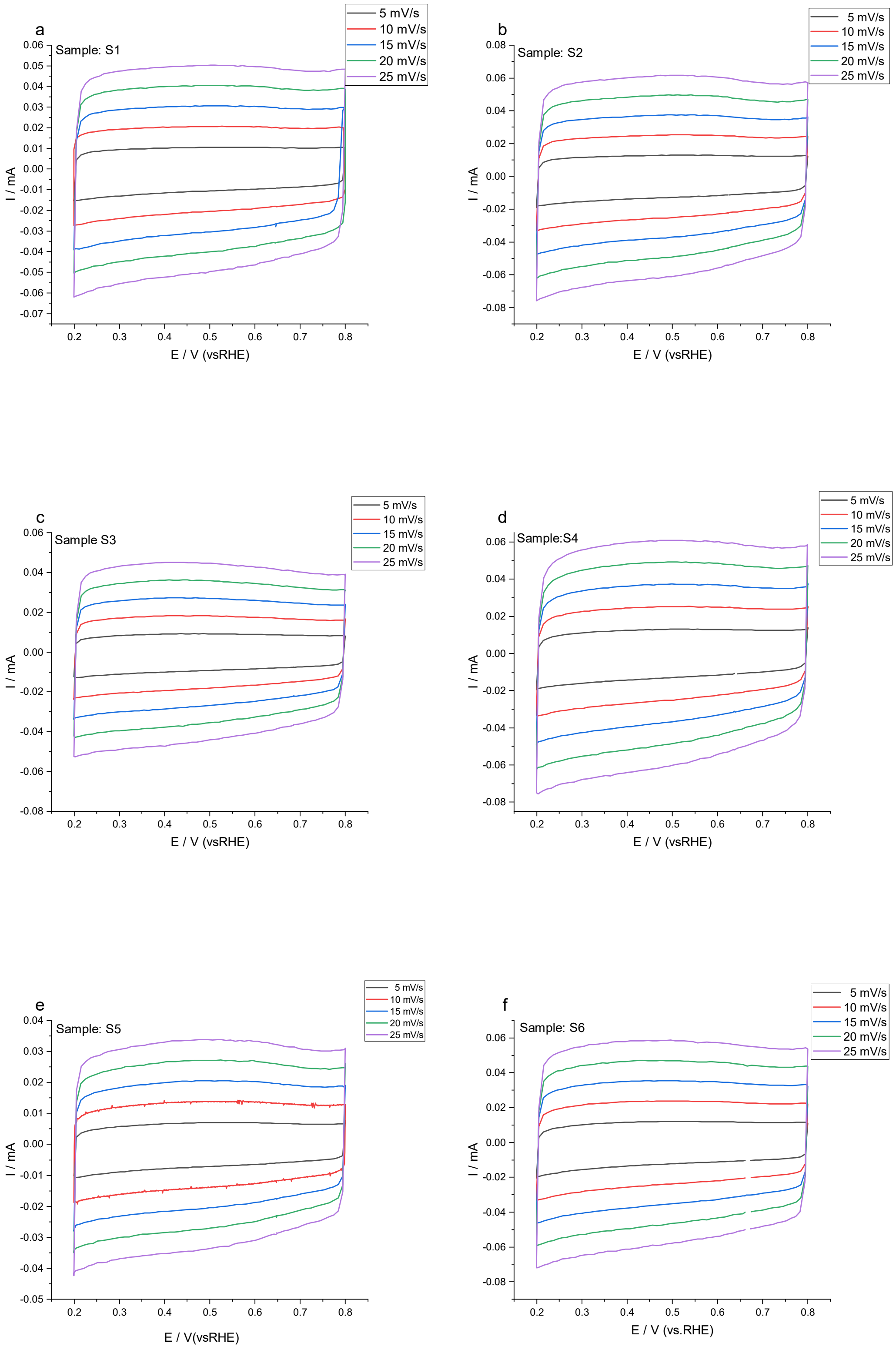

Figure SI1. *Cyclic voltammogram curves of samples S1 to S6 in the double layer region at scan rates of 5, 10, 15, 25 mV s^{-1} in 0.1 M $HClO_4$ aqueous electrolyte, under Ar saturation.*

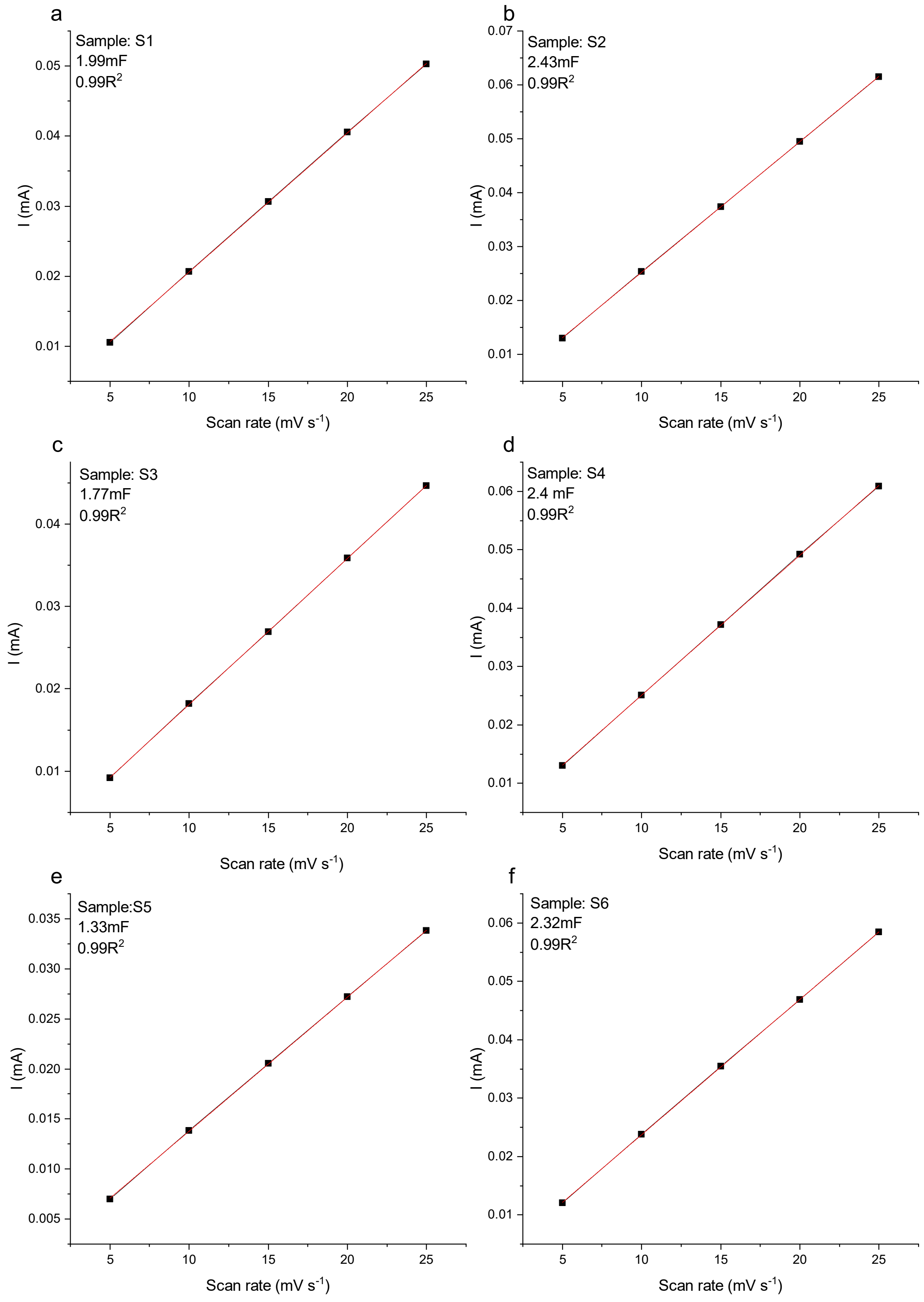

Figure SI2. *Current (taken at the potential of 0.5 V_{RHE}) as a function of scan rate derived from Figure SI1.*

Table SI1. *Faradaic efficiency, BET surface area and the partial current densities for H_2O_2 generation of the tested porous carbon-based samples at -0.2 V in 0.1 M $HClO_4$.*

Sample	FE @ -0.2 V [%]	$j_{H_2O_2}$@ -0.2 V [mA/$_{geom.}$cm^2]	S_{BET} [m^2/g]	ECSA [cm^2]	$j_{H_2O_2}$@ -0.2 V [µA/$_{EASA}$cm^2]
S1	55 ± 4	1.48	1594	99.5	2.76
S2	51 ± 3	1.70	1515	121.5	2.74
S3	58 ± 0.1	1.68	1354	88.5	3.76
S4	60 ± 3	1.84	1357	120	2.78
S5	63 ± 3	1.85	512	66.5	5.31
S6	69 ± 2	1.70	1677	116	2.87

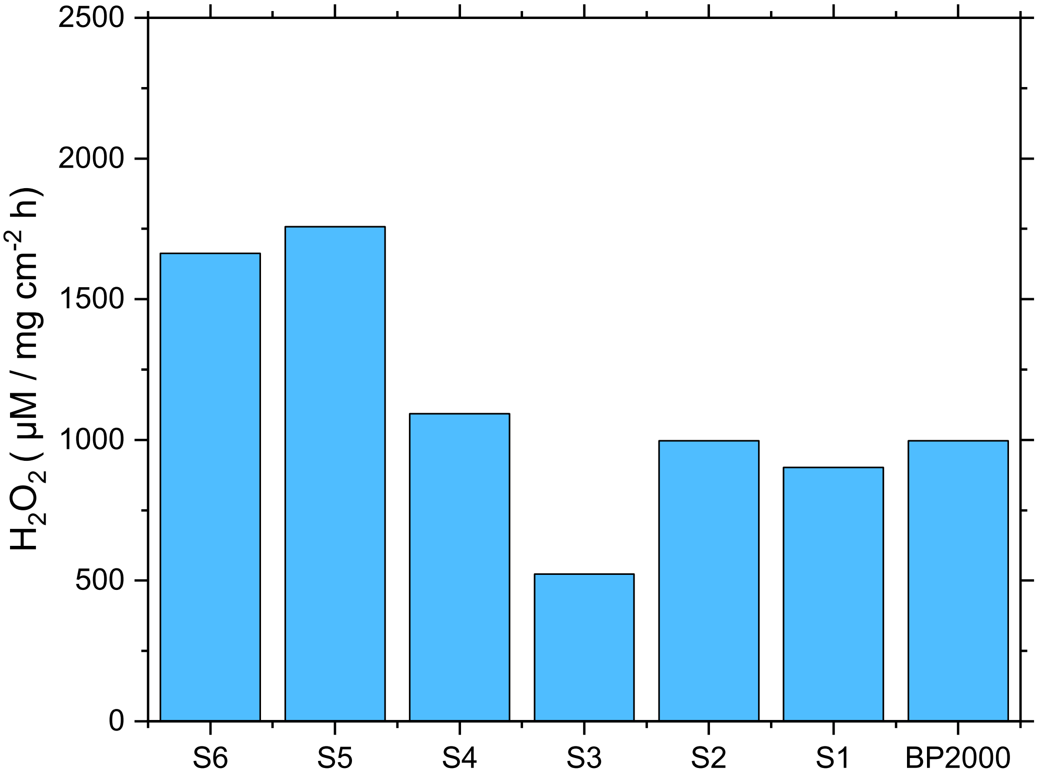

Figure SI3. *The concentration of H_2O_2 produced in a bulk electrolysis experiment, under continuous O_2 saturation after one hour normalized by the catalyst loading (0.1 mg/ cm^2); BP2000 as a direct comparison, measured under the same conditions and catalyst loading.*

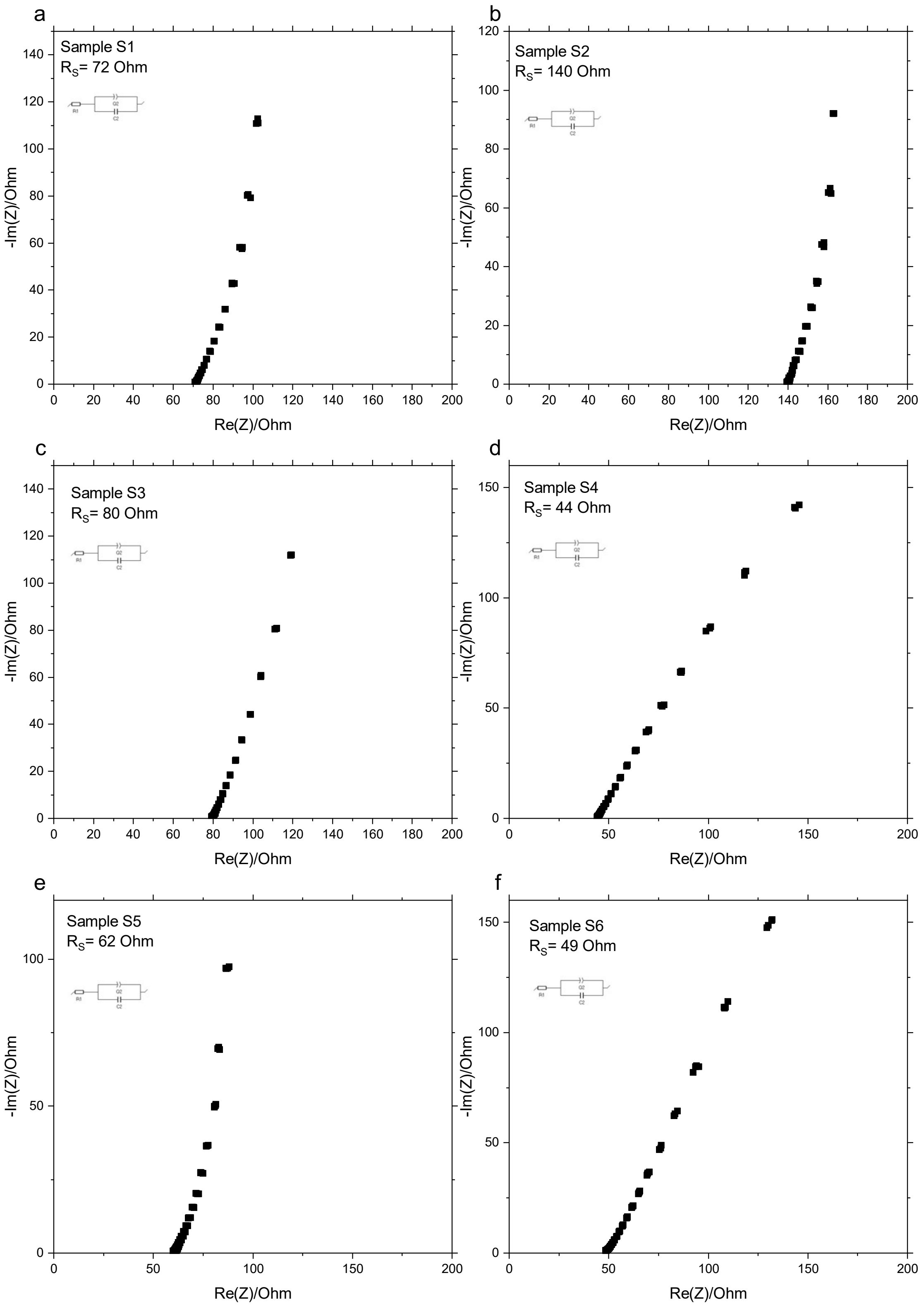

Figure SI4. *Nyquist plots of the samples S1 to S6 in an Ar-saturated in 0.1 M $HClO_4$ aqueous electrolyte.*

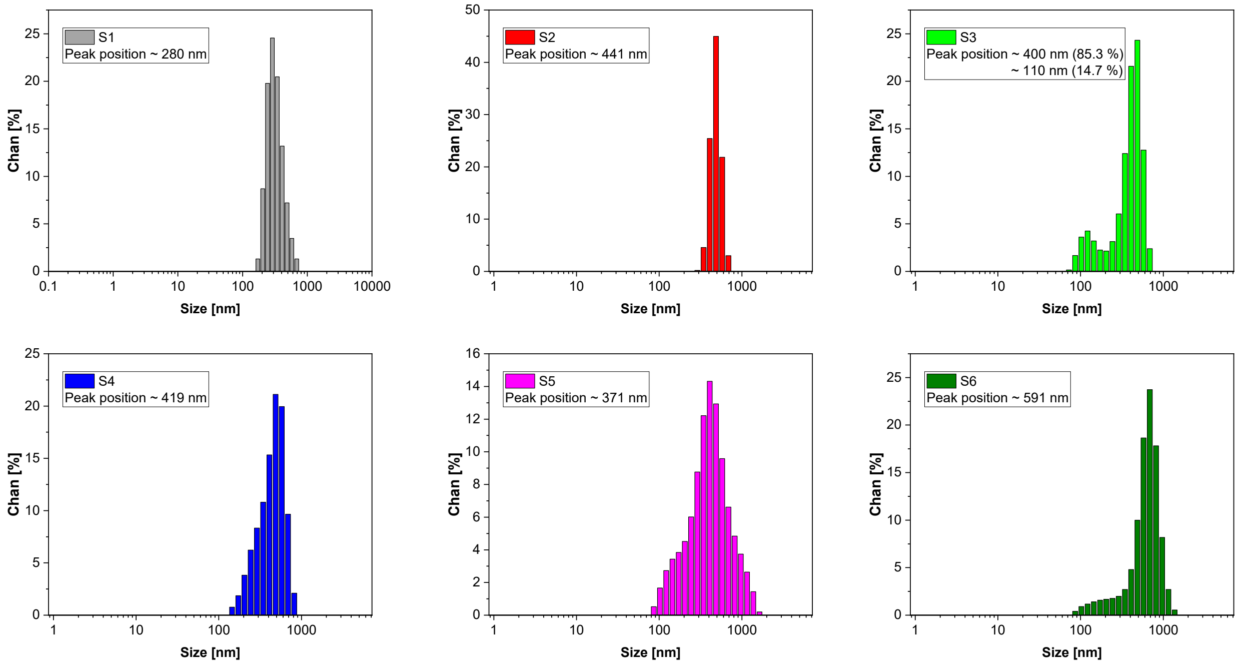

Figure SI5. *Particle size distribution of the carbon samples in 70% isopropanol – 30% water.*

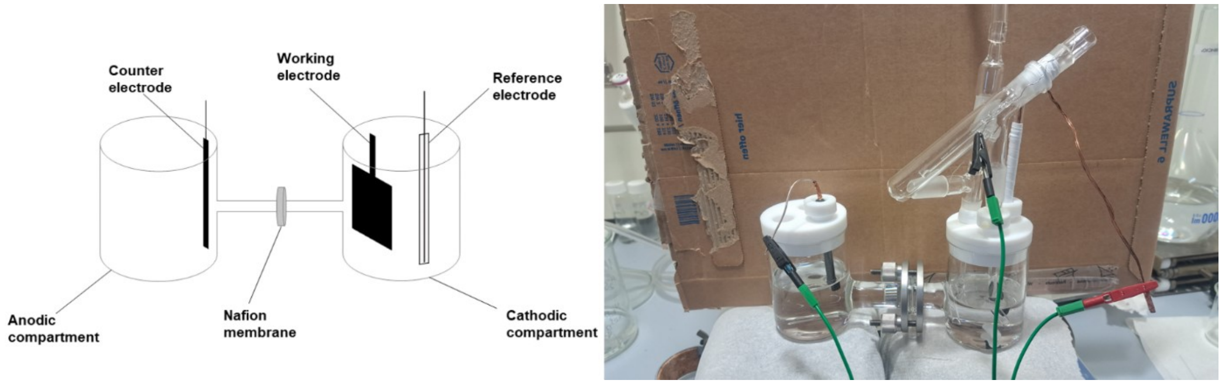

Figure SI6. *Schematic representation of the H-cell (left) and picture of the H-cell (right).*

A) B) C)

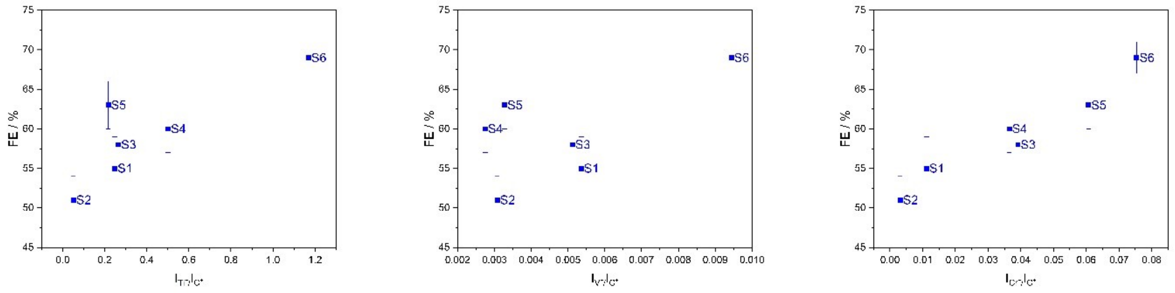

D) E) F)

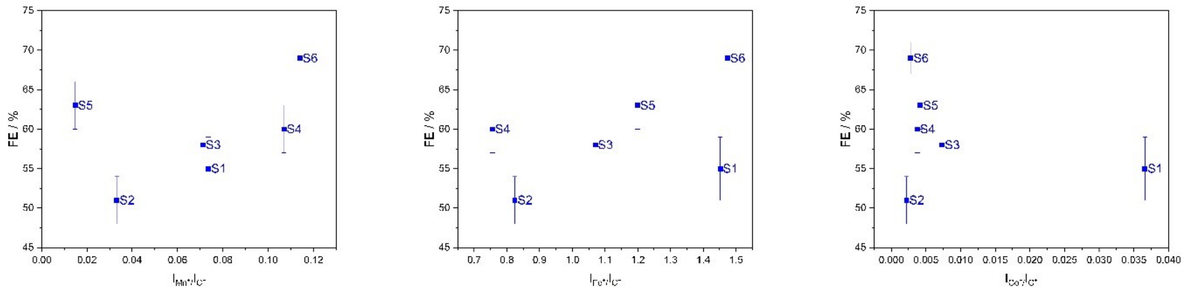

G) H) I)

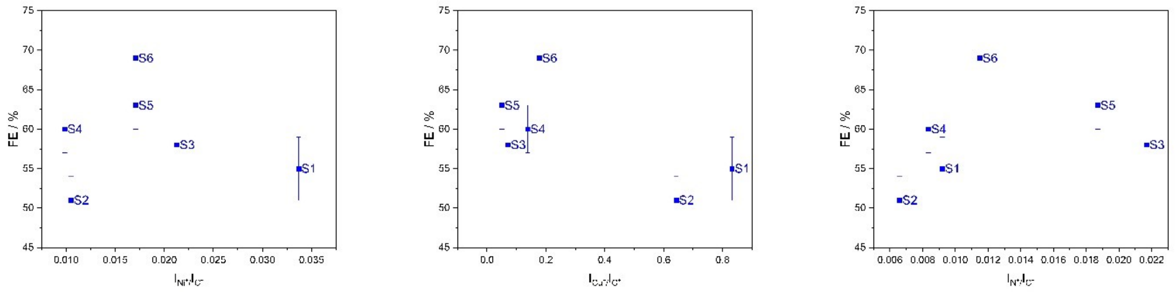

Figure SI7. *The Faradaic efficiencies of the different carbon materials as a function of the secondary ion intensities I_{M^+} of different transition metal elements M and the secondary ion intensity of the nitrogen I_{N^+}, normalized to the secondary ion intensity of the carbon mass peak I_{C^+}, as determined by TOF-SIMS for elements:* A) Ti, B) V, C) Cr, D) Mn, E) Fe, F) Co, G) Ni, H) Cu and I) N

Table SI2. *Elemental composition of samples S1 - S6 from XPS analysis. In addition, Na and K show minor concentrations in samples*

**Sample**	**C / at.%**	**O / at.%**	**N / at.%**	**Na / at.%**	**Si / at.%**	**K / at.%**
S1	75.7	19.1	2.4	0.2	2.6	0.0
S2	82.2	14.9	1.5	0.0	1.4	0.0
S3	84.0	10.0	4.0	0.0	1.1	0.9
S4	66.0	25.7	2.0	0.0	5.3	1.1
S5	85.1	9.6	3.8	0.0	1.5	0.0
S6	70.2	22.0	1.7	0.0	5.2	0.9

References:

1. Morales, D.M. and M. Risch, *Seven steps to reliable cyclic voltammetry measurements for the determination of double layer capacitance.* Journal of Physics: Energy, 2021. **3**(3): p. 034013.
2. Shi, H., *Activated carbons and double layer capacitance.* Electrochimica Acta, 1996. **41**(10): p. 1633-1639.
3. Vogel, A.I., et al., *Vogel's Textbook of Quantitative Chemical Analysis*. 2000: Prentice Hall.
